# CRL2^LRR-1^ E3-Ligase Regulates Proliferation and Progression through Meiosis in the *Caenorhabditis elegans* Germline

**DOI:** 10.1371/journal.pgen.1003375

**Published:** 2013-03-28

**Authors:** Julien Burger, Jorge Merlet, Nicolas Tavernier, Bénédicte Richaudeau, Andreas Arnold, Rafal Ciosk, Bruce Bowerman, Lionel Pintard

**Affiliations:** 1 Institut Jacques Monod, CNRS, UMR 7592, Université Paris Diderot, Sorbonne Paris Cité, Paris, France; 2 Friedrich Miescher Institute for Biomedical Research, Basel, Switzerland; 3 Institute of Molecular Biology, University of Oregon, Eugene, Oregon, United States of America; University of California Santa Cruz, United States of America

## Abstract

The ubiquitin-proteolytic system controls the stability of proteins in space and time. In this study, using a temperature-sensitive mutant allele of the *cul-2* gene, we show that CRL2^LRR-1^ (CUL-2 RING E3 ubiquitin-ligase and the Leucine Rich Repeat 1 substrate recognition subunit) acts at multiple levels to control germline development. CRL2^LRR-1^ promotes germ cell proliferation by counteracting the DNA replication ATL-1 checkpoint pathway. CRL2^LRR-1^ also participates in the mitotic proliferation/meiotic entry decision, presumably controlling the stability of meiotic promoting factors in the mitotic zone of the germline. Finally, CRL2^LRR-1^ inhibits the first steps of meiotic prophase by targeting in mitotic germ cells degradation of the HORMA domain-containing protein HTP-3, required for loading synaptonemal complex components onto meiotic chromosomes. Given its widespread evolutionary conservation, CUL-2 may similarly regulate germline development in other organisms as well.

## Author Summary

Maintenance of the germline depends on the presence of a germline stem cell pool with self-renewal potential that produces gametes upon meiotic differentiation. Factors regulating the balance between germline stem cell self-renewal and meiotic differentiation ensure germline homeostasis, whereas disruption of these regulatory mechanisms can lead to sterility or cancer. In this study, we show that the ubiquitin-proteolytic system (UPS), which selectively targets regulatory proteins for proteasomal degradation, controls germline development by acting at three different levels. The UPS promotes germ cell proliferation, regulates the balance between self-renewal and meiotic differentiation, and limits progression through meiotic prophase. In particular, we show that an E3 ubiquitin-Ligase nucleated around the cullin 2 (CUL-2) protein and using the Leucine rich repeat protein LRR-1 as substrate recognition subunit regulates in germ stem cells the stability of HTP-3, which is required for progression through meiotic prophase. These findings identify a previously unknown role for proteolytic regulation in germline development and also explain how the critical balance between germ cell proliferation and meiotic differentiation can be tightly and robustly controlled with multiple, parallel regulatory inputs.

## Introduction

The ubiquitin-proteolytic system has emerged as a central mechanism to regulate protein turnover spatially and temporally [1], [2]. In this system, ubiquitin, a small polypeptide of 76 amino acids, is covalently linked to a target protein through an enzymatic cascade, and the assembly of a poly-ubiquitin chain typically specifies that target protein for rapid degradation via the 26S proteasome [3]. The system can rapidly turn “off” regulatory proteins with high selectivity and is essential for numerous cellular processes such as transcription, signaling, DNA replication, DNA repair, cell cycle progression and differentiation. Not surprisingly, defects in the ubiquitin-proteolytic system have been implicated in a number of human diseases including cancers and neurodegenerative disorders [4]–[6].

An enzymatic cascade of three enzymes mediates the attachment of ubiquitin to substrate protein: the ubiquitin-activating enzyme (E1), ubiquitin-conjugating enzyme (E2), and ubiquitin-ligase (E3) [7]. Repeated cycles of ligation to the initial ubiquitin lead to poly-ubiquitination. The assembly of poly-ubiquitin chains can occur at different lysine residues within ubiquitin, with conjugation at lysines 11 and 48 typically leading to proteasomal degradation [8]–[11]–[12].

The paramount regulatory step in the cascade is the selective recognition of substrates, which is achieved by the E3-Ligase. Many E3 enzymes are nucleated around cullin scaffold subunits, with at least five cullin family members conserved in all metazoans [13], [14]. Each cullin scaffold nucleates multiple E3-ligase complexes that all contain a similar catalytic core but use different substrate recognition modules to engage their cognate substrate(s) [15], [16]. Over the past several years, Cullin RING E3-ligases nucleated around the cullins 1, 3 and 4 have been investigated extensively. By comparison, the functions of CRL2 complexes are relatively less well understood, with the noticeable exception of the CRL2^Von Hippel Lindau (VHL)^ complex that regulates the hypoxic response (for review see [17]). A Cul2 knockout mouse has not been reported and the roles of Cul2 during vertebrate development remain largely unknown.

**Figure 1:**
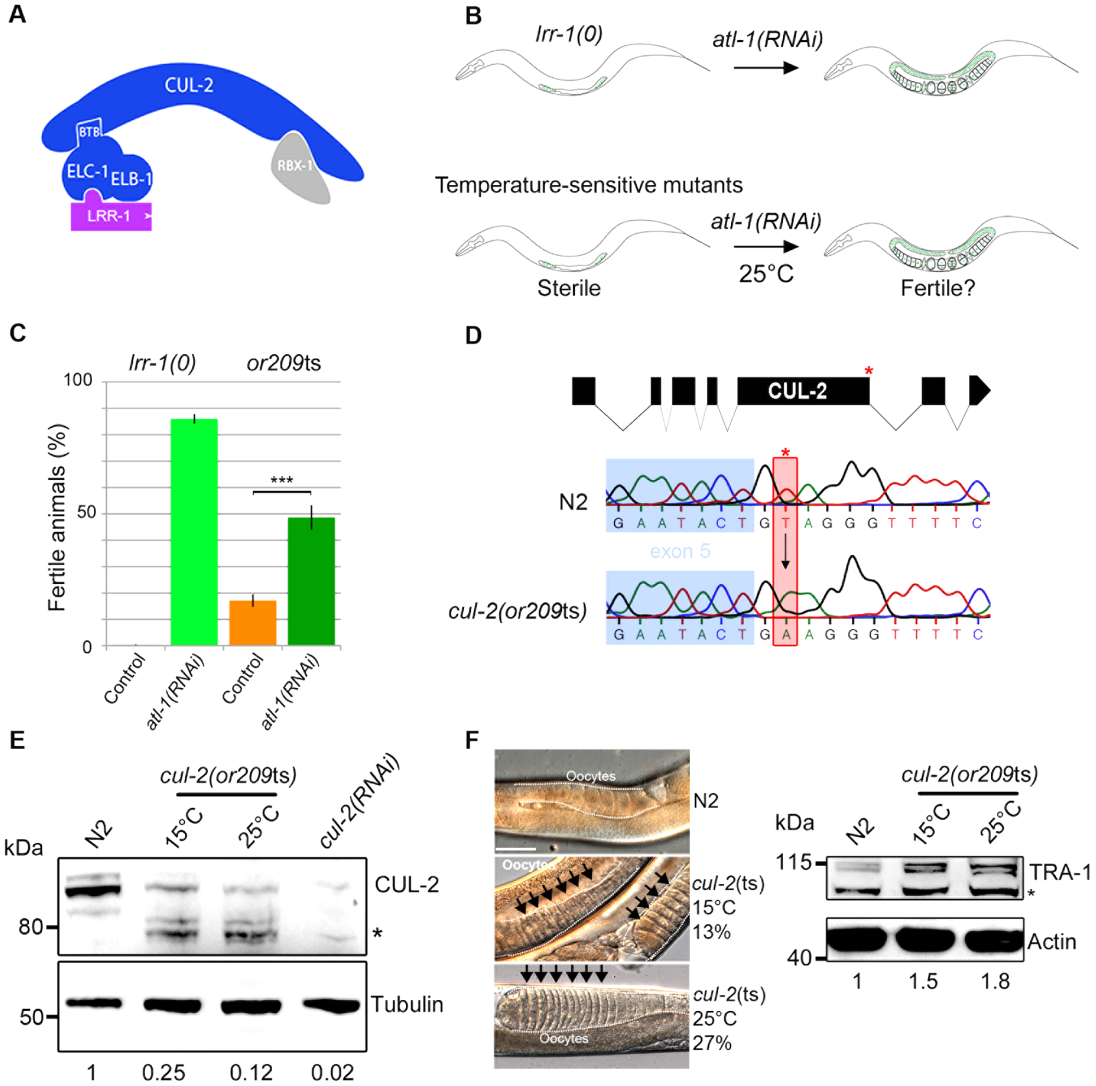
*or209* is a conditional *cul-2* temperature-sensitive allele. A- Schematic representation of the CRL2^LRR-1^ E3-ligase. This complex is composed of two modules nucleated around the CUL-2 subunit (arc-shaped in blue): the substrate recognition module and the catalytic site. The substrate recognition module comprises the adaptor protein ELC-1, ELB-1 (blue) and the LRR-1 substrate recognition subunit (purple), whereas the catalytic module contains the RING finger protein RBX-1 (grey). B- Schematic drawing of the RNAi-based screen used to search for temperature-sensitive (ts) alleles affecting the function of the CRL2^LRR-1^ complex. *lrr-1(tm3543)* mutant animals *(lrr-1(0))* are sterile but recover fertility upon *atl-1* depletion by RNAi (upper panel). Therefore, we searched for ts mutants that are sterile at 25°C but recover fertility upon inactivation of *atl-1*. The *or209*ts mutant fulfilled these criteria (lower panel). C- The *or209*ts mutation phenocopies inactivation of the CRL2^LRR-1^ complex. Graph showing the percentage of fertile *or209* animals (25°C) after control (orange bars) and RNAi-mediated depletion of *atl-1* (green bars). An average of eight different experiments is presented with 30 animals analysed in each experiment. D- Structure of the *cul-2* gene (upper panel); red asterisk depicts the location of the *or209* mutation. Chromatograms showing the T to A transversion found in the *cul-2*(*or209*ts) mutant (lower panels). E- Embryonic extracts of the indicated gentotype were separated by SDS-PAGE and immunoblotted with CUL-2 (upper panel) and tubulin (lower panel) antibodies (loading control). The asterisk marks the position of truncated forms of CUL-2 that probably lack the C-terminal part of the protein. The value at the bottom is the ratio between CUL-2 and tubulin signal intensities. The wild-type value was arbitrary defined as 1. F- The sex determination factor TRA-1 accumulates in *cul-2*(*or209*ts) mutant animals, presumably causing the feminisation of the germline. Micrographs of adult worms of the indicated genotypes analysed by DIC microscopy (left panel). The germline is outlined. Note the accumulation of packed oocytes (black arrows) in *cul-2*(*or209*ts) animals, which is characteristic of a feminisation (fem) phenotype. This phenotype is observed in 13% (n = 551) of animals maintained at 15°C and in 27% (n = 228) of animals shifted during 20 hours at 25°C from the L3 stage, but is never observed in wild-type (n>200). Right panel: worm extracts of synchronised *cul-2*(*or209*ts) animals cultivated at 15°C or shifted 20 hours at 25°C from the L3/L4 stage were separated by SDS-PAGE and blotted with TRA-1 (upper panel) antibodies. The asterisk marks the position of a non-specific band. The value at the bottom is the ratio between TRA-1 and actin signal intensities. The wild-type value was arbitrary defined as 1.

In *Caenorhabditis elegans*, CUL-2 is highly expressed in the germline and in early embryos, where it regulates numerous developmental processes including germ cell proliferation [18]–[20], sex determination [20], progression through oocyte meiosis [21], [22], cell polarity [21], cell fate determination [23] and cell cycle progression [19]. CUL-2 accomplishes these various functions by recruiting, via the adaptor protein elongin C (ELC-1), distinct substrate-recognition subunits (SRS) that specifically engage their substrates (Figure 1A) [17]. Several SRS have been identified in *C. elegans*, though in most cases their relevant targets remain to be identified. For instance, the Leucine Rich Repeat protein LRR-1 is an SRS expressed throughout the germline where it is essential for germ cell proliferation [19], but the precise function of the CRL2^LRR-1^ E3-Ligase in the germline is still poorly understood.

We report here the isolation of a temperature-sensitive mutation in the *C. elegans cul-2* gene. The *cul-2*(*or209*ts) mutation recapitulates all the *cul-2(RNAi)* and *cul-2(0)* phenotypes and also reveals several novel functions in the *C. elegans* germline. More specifically, we show that besides promoting germ cell proliferation, CUL-2 has at least two other functions in the germline: it participates in the proliferation versus meiotic entry decision and inhibits the first steps of meiotic prophase. Furthermore, we show that the CRL2^LRR-1^ E3-ligase inhibits meiotic prophase progression at least in part by promoting degradation of HTP-3, a HORMA domain containing protein. HTP-3 is required for progression through meiotic prophase and for the assembly of the synaptonemal complex, the zipper-like structure that tethers homologous chromosomes together.

## Results

### Identification of a temperature-sensitive allele in the *cul-2* gene: *cul-2*(*or209*ts)

The evolutionarily conserved CRL2^LRR-1^ E3-ligase is essential for germline development in *C. elegans*: *lrr-1(0)* and *cul-2(0)* null mutants are sterile with a small germline [18], [19]. However, the precise function of this enzyme is still poorly understood as the sterility in null mutants limits analysis of gene requirements. To circumvent this problem, we screened for temperature-sensitive (ts) mutants that resemble *lrr-1(0)* mutants. While *lrr-1(0)* animals are sterile, this phenotype is suppressed by inactivation of the DNA replication ATR/ATL-1 checkpoint pathway [19]. Therefore, we screened for ts mutants that, like *lrr-1(0)* mutants, are sterile at the restrictive temperature of 25°C but recover fertility upon *atl-1* depletion by RNAi (Figure 1B). Such mutants might be specifically defective in the function of the CRL2^LRR-1^ complex and allow for a more careful analysis of gene requirements in the germline at different temperatures that only partially compromise E3-ligase function.

After screening a collection of ts mutants (Figure S1, Table S1) we found one that fulfilled the above criteria: *or209*ts. Like *lrr-1(0)* mutants, most *or209*ts mutant animals are sterile at 25°C but recover fertility upon *atl-1* inactivation by RNAi (Figure 1C). *or209*ts has previously been linked to chromosome III [24], and we used single nucleotide polymorphisms to further map the *or209*ts mutation to the end of this chromosome, which contains the cullin gene *cul-2* (21.36 cM) (Figure S1). We next found that *or209*ts fails to complement a known *cul-2* null allele (material and methods), and we then sequenced the *cul-2* gene from *or209*ts genomic DNA and found a single nucleotide change relative to wild-type in the splicing donor site at the fifth exon-intron boundary (Figure 1D). This mutation substantially reduces CUL-2 protein levels even at the permissive temperature (15°C), but this effect is aggravated at the restrictive temperature (25°C) (Figure 1E). Consistent with the partially conditional effect on protein levels, *cul-2*(*or209*ts) animals present high levels of embryonic lethality (30%) and a severe reduction in brood size even at the permissive temperature of 15°C, compared to wild-type (Figure S1). Further analysis revealed that *cul-2*(*or209*ts) animals present typical *cul-2* loss-of-function phenotypes, including the accumulation of abnormally high levels of the TRA-1 protein, presumably causing feminization of the germline (Figure 1F), and of the PIE-1 protein (Figure S2), both well-defined targets of CRL2^FEM-1^ and CRL2^ZIF-1^ E3-ligases, respectively [23], [25]. We conclude that *or209*ts is a conditional allele of *cul-2*. To our knowledge, *or209*ts is the first temperature-sensitive allele yet identified that affects a metazoan cullin gene.

### CRL2^LRR-1^ E3-ligase promotes germ cell proliferation in adult germlines

**Figure 2:**
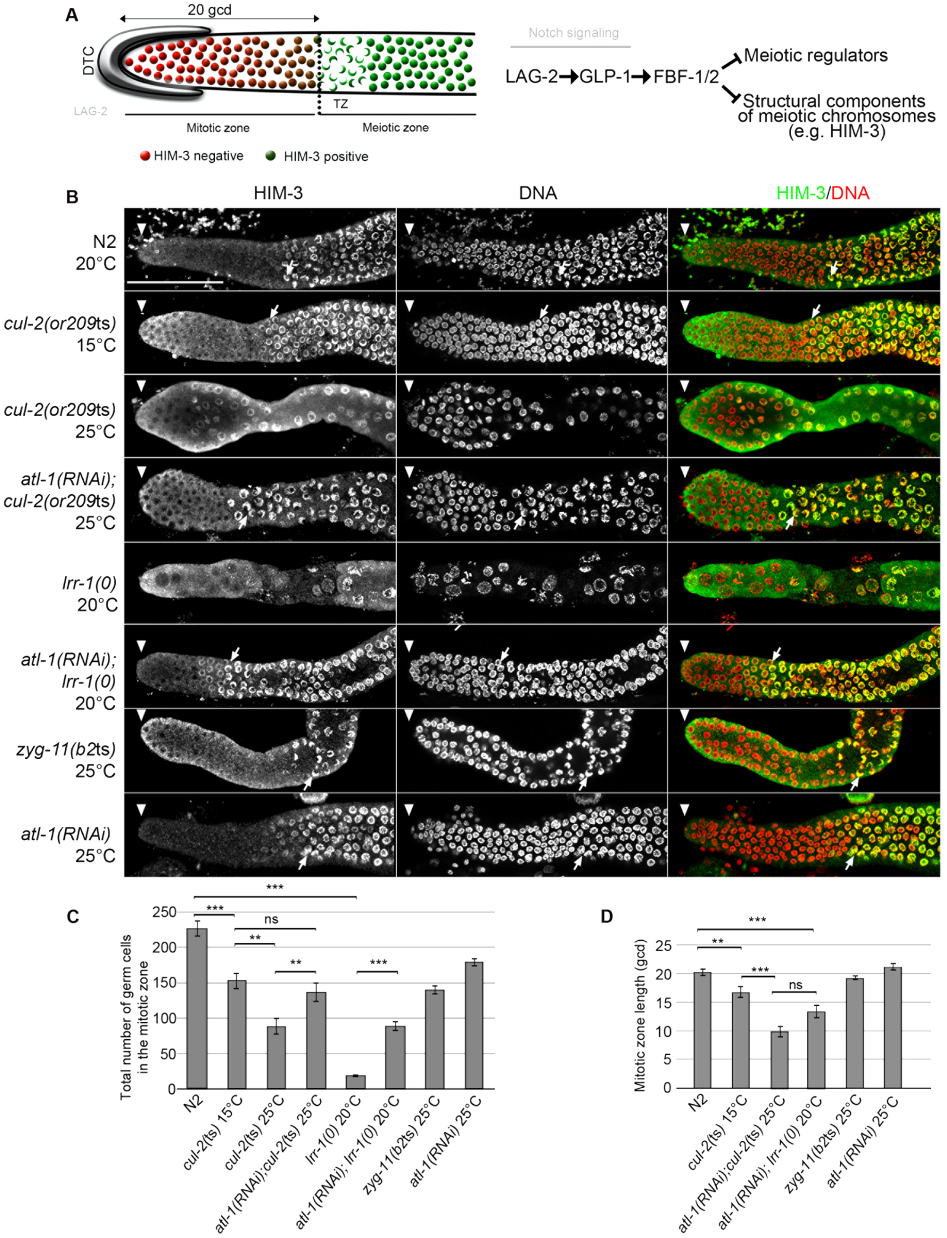
CRL2^LRR-1^ E3-ligase promotes germ cell proliferation. A- Schematic drawing of an adult distal germline. The distal tip cell (DTC) niche is located at the distal end. The DTC expressed the Notch ligand LAG-2, which is shown in grey. The mitotic zone contains germ cells in the mitotic cell cycle (red), including a germline stem cell (GSC) pool distally and possibly transit-amplifying germ cells proximally; at its proximal edge, some germ cells have entered pre-meiotic S-phase. The transition zone contains germ cells in meiotic S-phase and in meiotic prophase (nuclear crescents) that start to accumulate HIM-3 (green), a marker of meiotic prophase. The dashed line marks the boundary between the mitotic and transition zones (left panel). A simplified pathway with key regulators of the balance between GSC renewal and meiotic differentiation is shown in the right panel. Positive regulation (arrows) and negative regulation (bars). B- Representative images of dissected gonads of animals of the indicated genotypes taken 24 hours after the mid-L4 stage stained with HIM-3 antibodies (green) and DAPI (red). The distal end of the germline (arrowhead) is oriented toward the left, and the proximal end is oriented toward the right in this and other figures. Arrow, nuclei with crescent shape that is typical of nuclei in meiotic prophase. Germlines in each panel were treated identically and fluorescent images taken at the same settings. Scale bars: 50 µM. C- Graphs showing the mean of the total number of germ cells in the mitotic zone of the germline and D- the quantification of the size of the mitotic zone, in germ cell diameters (gcd) from the distal end in animals of the indicated genotypes. At least ten germlines of each genotype were scored.

Using the *or209*ts allele, we re-examined CUL-2 function in the germline. The *C. elegans* germline is spatially organized and contains - from the distal to proximal end -mitotically proliferating stem cells, meiotic germ cells, and gametes (sperm or oocyte). The mitotic zone extends 18–20 germ cell diameters (gcd) along the gonadal axis from the distal end, with the total number of germ cell nuclei exceeding 200 [26] (Figure 2A). In the transition zone (TZ), just proximal to the mitotic zone, cells enter into meiotic prophase [27], [28]. At this stage, structural components accumulate on meiotic chromosomes (e.g HIM-3 [29]) such that germ cell nuclei present a characteristic crescent shape that is readily evident by DAPI staining [30].

Corroborating our previous observations indicating that loss of the CRL2^LRR-1^ E3-ligase results in a cell cycle arrest [19], the number of germ cells in the mitotic zone was severely reduced and nuclei were enlarged in *cul-2*(*or209*ts) mutants raised from early larval stages (L1) at the restrictive temperature of 25°C. As expected, this phenotype was largely suppressed by inactivation of the ATL-1/DNA replication pathway (Figure 2). In addition, we noticed that the size of the mitotic zone was reduced in *cul-2*(*or209*ts) animals, as determined by scoring the distance, in gcd, between the distal end of the germline and the appearance of both nuclear crescents and HIM-3 positive cells (Figure 2B, arrows and 2C–D). The mitotic zone was reduced to 15 gcd in *cul-2*(*or209*ts) mutants raised to adulthood at permissive temperature (15°C), and even more reduced in mutants raised at the restrictive temperature (25°C). However, it was difficult to rigorously quantify the phenotype at 25°C because germ cell nuclei were both enlarged and reduced in number (Figure 2). We thus analyzed the size of this region in *atl-1(RNAi)*; *cul-2*(*or209*ts) mutant hermaphrodites and also observed a smaller mitotic zone in these animals. The size of the mitotic zone was similarly reduced in *atl-1(RNAi)*; *lrr-1(0)* animals but was unaffected in the control *atl-1(RNAi)* (Figure 2B and 2D). Finally, ZYG-11 is another substrate-recognition subunit of a CRL2 complex that appears dispensable in germline stem cells [31], and accordingly, the size of the mitotic zone was unaffected in *zyg-11*(*b2*ts) mutant germlines at 25°C (Figure 2B and 2D). Collectively, these results indicate that CUL-2, in combination with LRR-1, promotes germ cell proliferation by counteracting the ATL-1/DNA replication checkpoint but may also influence the size of the mitotic zone independently of ATL-1. Indeed, *atl-1* depletion in *lrr-1* and *cul-2* mutants does not fully rescue the size of the mitotic zone and *atl-1* depletion in WT animals does not affect the size of the mitotic zone.

### *cul-2* participates in the proliferation versus meiotic entry decision

**Figure 3:**
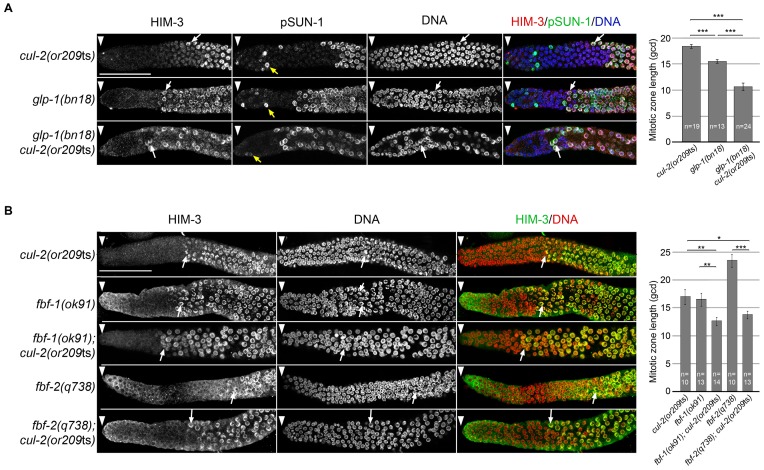
A- Representative images of dissected gonads of animals of the indicated genotypes, maintained at 20°C from L1 and taken 24 hours after L4, stained with HIM-3 (red), SUN-1 Ser8-Pi (green) antibodies and with DAPI (blue) (left panel). Arrowhead, distal end of the germline. Yellow arrow, germ cells in G2-M phase of the cell cycle and white arrow, nuclei with crescent-shape morphology typical of meiotic prophase. Quantification of the size of the mitotic zone in germ cell diameters from the distal end is shown on the right. B- Representative images of dissected gonads of animals of the indicated genotypes maintained at 20°C from L1 and taken 24 hours after the L4 stage stained with HIM-3 antibodies (green) and counterstained with DAPI (red) (left panel). Arrowhead, distal end of the germline. Arrow, nuclei with crescent-shape morphology typical of meiotic prophase. Quantification of the size of the mitotic zone in germ cell diameters from the distal end is shown on the right. Note that although *fbf-1* and *fbf-2* genes are largely redundant for promoting mitosis, they have opposite roles in regulating the size of the mitotic region. The mitotic region is smaller than normal in *fbf-1(0)* mutants but larger than normal in *fbf-2(0)* mutants. This opposite role is largely due to different spatial regulation and reciprocal 3′UTR repression; *fbf-2* expression is limited to the distal germline whereas *fbf-1* expression is much broader [58].

We thus investigated whether *cul-2*, in addition to regulating mitotic proliferation, might also have a role in preventing meiotic entry, by looking for genetic interactions between *cul-2* and genes regulating the proliferation versus meiotic entry decision. GLP-1/Notch signaling controls the decision between self-renewal and entry into the meiotic cell cycle [28]. Downstream of Notch, the nearly identical Puf (*Pu*milio and *F*BF)-domain RNA-binding proteins FBF-1/2 prevent meiotic entry by repressing the translation of meiotic promoting factors and structural components of meiotic chromosomes (Figure 2A) [32], [33]. We therefore constructed double mutants between *cul-2*(*or209*ts) and *glp1(bn18)*, a temperature-sensitive *glp-1* mutant in which the Notch signaling pathway is partially defective [34], and between *cul-2*(*or209*ts) and *fbf-1(0)* or *fbf-2(0)*, and scored meiotic entry in the double versus single mutants at the semi-permissive temperature of 20°C. At this temperature, germ cell proliferation is only modestly affected in *cul-2*(*or209*ts) mutants (Figure 3A).

To score meiotic entry in these different mutant backgrounds, we used a combination of cellular morphology (nuclear crescents) and molecular markers. More specifically, we monitored the appearance of HIM-3 and SUN-1 Ser8-Pi (P-SUN-1) on meiotic chromosomes [35]. P-SUN-1 is first detected in the mitotic zone, at nuclear periphery, in germ cells that are in mitosis from prometaphase onward (Figure 3A, yellow arrows), and then in meiosis, in the transition zone (TZ) at foci and patches, as well as over the nuclear envelope, as reported previously [35]. As shown in Figure 3A, the *cul-2ts* mutant enhanced the premature meiotic entry phenotype of the *glp-1(bn18)* mutant, as evidenced by the premature meiotic entry in the *glp-1(bn18) cul-2*(*or209*ts) double mutants, compared to the single mutants. Likewise, when combined with *cul-2*(*or209*ts), both *fbf-1* and *fbf-2* mutants showed a smaller mitotic zone than single mutants (Figure 3B) indicating that *cul-2* influences the size of the mitotic zone. Collectively, these results indicate that CUL-2 acts with the Notch signaling pathway and FBF-1/2 to prevent meiotic entry.

### CRL2^LRR-1^ regulates HTP-3 stability in the mitotic zone of the germline

#### CRL2^LRR-1^ acts through HTP-3 in the *C. elegans* germline

Entry into meiosis requires the coordination of a number of events: upon exit from the mitotic zone, germ cells initiate the chromosome dynamics required for meiotic pairing and synapsis. In preparation for this transition, proximal cells in the mitotic zone activate the expression of both regulators of meiotic entry and chromosomal proteins required for synapsis (e.g HIM-3) that are then recruited on meiotic chromosomes. CUL-2 may thus inhibit meiotic entry by controlling the stability of regulators of meiotic entry in the mitotic zone of the germline.

**Figure 4:**
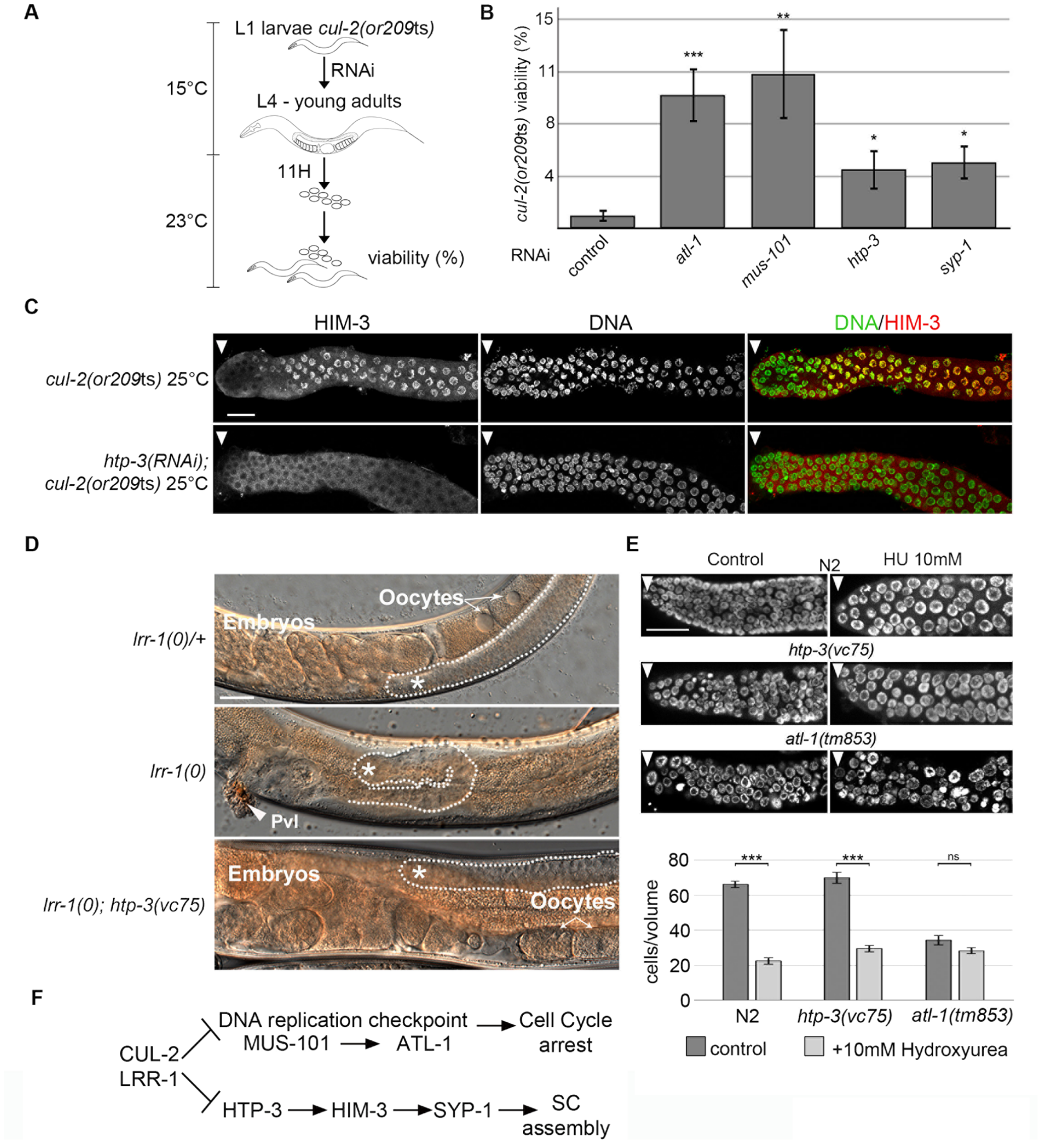
CRL2^LRR-1^ E3-ligase acts through HTP-3 in the *C. elegans* germline. A- Flow-chart of the *cul-2*(ts) RNAi-based suppressor screen. At 15°C, synchronised *cul-2*(ts) L1 larvae were fed with bacteria expressing dsRNA, then were shifted to a semi-permissive temperature (23°C) at the L4 stage. After 11 hours, young adults were removed, and embryonic viability was determined and plotted. B- Graphs showing the percent viability of *cul-2*(*or209*ts) animals after RNAi depletion of the indicated genes. C- Representative images of dissected gonads of the indicated genotypes stained with HIM-3 antibodies (red) and DAPI (green) are shown. Scale bars: 50 µM. Arrow marks the distal end of the germline D- Reduction of *htp-3* function suppresses *lrr-1(0)* mutant animal sterility. Micrographs of adult worms of the indicated genotypes were analysed by DIC microscopy. Thirty-four percent of *lrr-1(0)*; *htp-3(vc75)* double mutants are fertile (n = 112). The germline is outlined, and the presence of oocytes and embryos is indicated. The asterisk marks the distal region of the germline. E- The DNA replication checkpoint is functional in *htp-3(vc75)* mutant animals. Animals of indicated genotypes were exposed to 10 mM Hydroxyurea (HU) and germlines were dissected 24 h post-L4 and stained with DAPI (upper panel). The number of germ nuclei in a given volume was determined and plotted (bottom panel). Note that HU treatment of wild-type (N2) animals leads to S-phase arrest that manifests as enlarged nuclei with an overall reduction in the number of nuclei in the mitotic zone of the germline. HU treatment similarly arrested *htp-3(vc75)* mutant germ cells, whereas checkpoint deficient *atl-1(tm853)* mutant germ cells fail to arrest cell cycle progression, indicating that the DNA replication checkpoint is functional in the *htp-3(vc75)* mutant. F- Pathway diagram summarizing the observed genetic interactions. CUL-2 and LRR-1 counteracts the DNA replication checkpoint pathway, which is composed of MUS-101 and ATL-1, and inhibits HTP-3, which promotes the recruitment of HIM-3, SYP-1 and the assembly of the synaptonemal complex (SC). Positive regulation (arrows) and negative regulation (bars).

Interestingly, an RNA interference (RNAi)-based screen (JM and LP, unpublished data) for suppressors of *cul-2*(*or209*ts) lethality at a semi-permissive temperature (23°C) identified, in addition to ATL-1 and its regulator MUS-101/TopBP1 [36], the axial element component HTP-3, which is required for the progression through meiotic prophase [37], [38], and SYP-1, a core component of the synaptonemal complex (SC) [39] (Figure 4A and 4B).

HTP-3 belongs to the family of HORMA (Hop1-Rev1-Mad2) domain-containing proteins [40], and is required for preparing chromosomes for meiosis. More specifically, HTP-3 is required for loading on chromosomes the meiotic cohesin REC-8 and the axial and transverse components of the synaptonemal complex, including HIM-3 and SYP-1; for the formation of double-strand breaks that initiate meiotic recombination; and for implementation of the meiotic DNA damage ATM-1/checkpoint [38], [41], [42].

Given that HTP-3 promotes HIM-3 and SYP-1 loading on chromosomes, we investigated the potential role of CRL2^LRR-1^ in HTP-3 regulation. As expected, the accumulation of HIM-3 on chromosomes in *cul-2*(*or209*ts) germ cells depends on HTP-3 because HIM-3 failed to accumulate on chromosomes in *htp-3(RNAi)*; *cul-2*(*or209*ts) mutant germlines (Figure 4C). To determine whether LRR-1 acts through HTP-3 in the germline, we asked whether *htp-3* reduction-of-function suppressed *lrr-1(0)* mutant sterility. To this end, we took advantage of the *htp-3(vc75)* allele, which only modestly compromises HTP-3 function [42], and constructed *lrr-1(tm3543)*; *htp-3(vc75)* double mutant animals. Importantly, a significant fraction (34%, n = 115) of *htp-3(vc75)*; *lrr-1(tm3543)* double mutant animals were fertile and produced embryos, in contrast to the highly penetrant sterility phenotype observed in *lrr-1(tm3543)* single mutants (Figure 4D). The observed suppression of the *lrr-1(tm3543)* sterility by the *htp-3(vc75)* allele is not merely resulting from an inactive ATL-1 checkpoint pathway in the *htp-3(vc75)* mutant because the DNA replication checkpoint is fully functional in this mutant (Figure 4E and Figure S3). Collectively, these results indicate that LRR-1 acts through HTP-3 in the germline (Figure 4F).

#### CRL2^LRR-1^ and the 26S proteasome control HTP-3 levels in the mitotic zone of the germline

**Figure 5:**
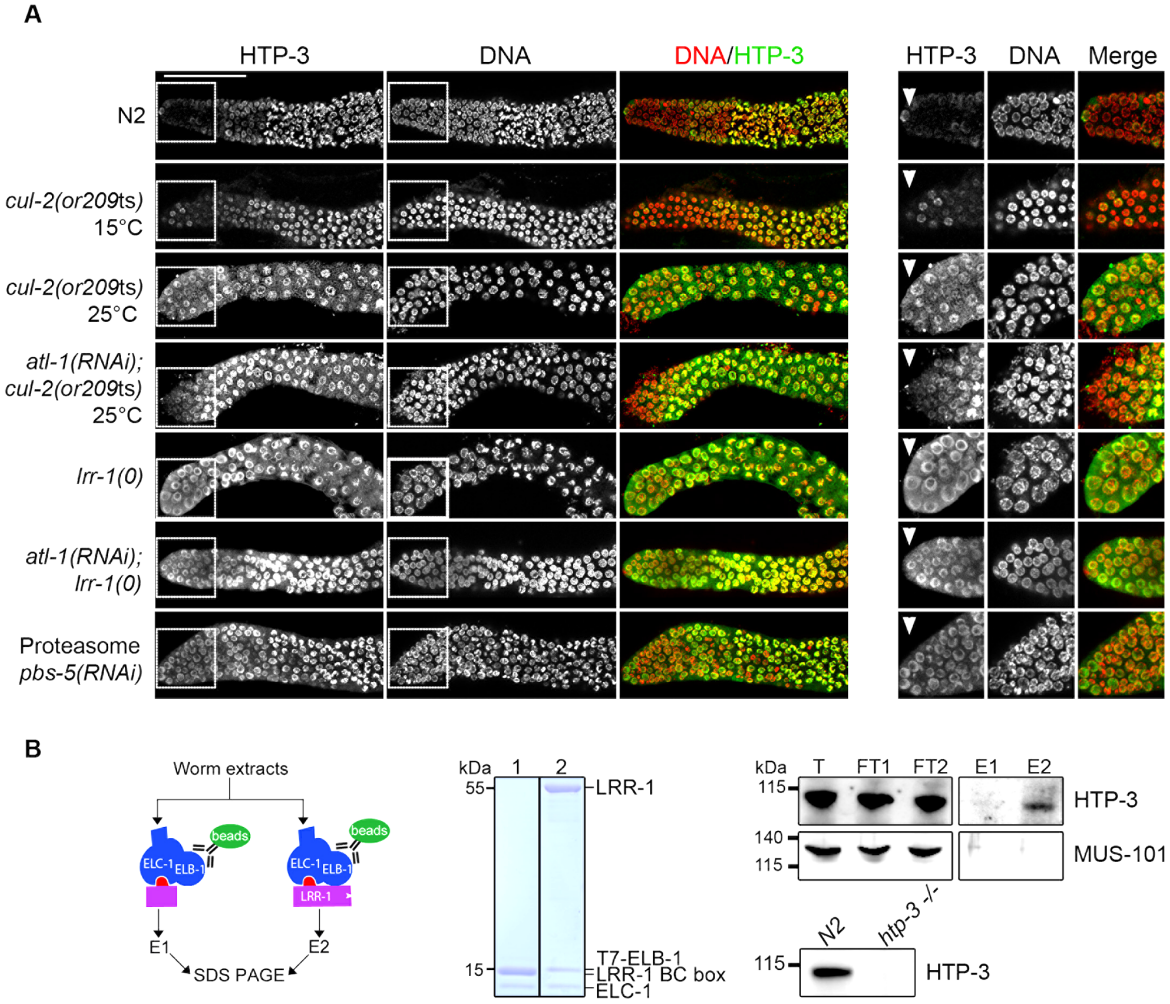
CRL2^LRR-1^ E3-ligase regulates HTP-3 levels in the mitotic zone of the germline. A- HTP-3 accumulates to high levels in *cul-2*(*or209*ts), *lrr-1(0)* and *pbs-5(RNAi)* germlines. Representative images of dissected gonads of the indicated genotypes stained with HTP-3 (green) antibodies and DAPI (red) (left panel). Insets show germ cells located in the most distal region of the germline (right panel) B- HTP-3 physically interacts with LRR-1. A flow chart of the experiment is presented in the left panel. Briefly, total worm extracts were incubated with the recombinant 6xHis-LRR-1/ELC-1/T7-ELB-1 trimeric complex immobilised on T7-agarose beads (green oval). As a control, we used a trimeric complex in which the substrate-binding interface LRR-1 (purple rectangle) was deleted. The middle panel shows a Coomassie-stained gel of the recombinant trimeric complexes containing either truncated or full-length LRR-1 protein (lane 1 and 2 respectively). Right panel: the trimeric complexes and associated proteins were eluted with loading buffer and analysed by western blot using MUS-101- and HTP-3-specific antibodies. T, total; FT, Flow-Through; E, elution. Lower right: total extracts of N2 and *htp-3(0)* worms were separated by SDS-PAGE and immunoblotted with HTP-3 antibodies.

To test whether CRL2^LRR-1^ regulates HTP-3 stability, we assessed HTP-3 protein levels by immunostaining *cul-2*(*or209*ts) and *lrr-1(0)* mutant germlines. In control germlines, HTP-3 was expressed at low levels in the most distally located germ cells and at high levels on chromosomes in the most proximal mitotic germ cells (Figure 5A) [38], [43]. In contrast to wild-type, HTP-3 was abundant in the most distally located germ cells in both *cul-2*(*or209*ts) and *lrr-1(tm3543)* mutants (Figure 5A). HTP-3 similarly accumulates in *atl-1(RNAi)*; *cul-2*(*or209*ts) and *atl-1(RNAi)*; *lrr-1(tm3543)* germlines, indicating that down-regulation of the DNA replication checkpoint does not affect HTP-3 levels. Importantly, HTP-3 also accumulates in the distal region after inactivation of PBS-5, a proteasome subunit, suggesting that CRL2^LRR-1^ may target HTP-3 for degradation (Figure 5A).

To ask whether the negative regulation of HTP-3 by CRL2^LRR-1^ E3 ligase involves physical interaction between the two proteins, we incubated total worm extracts with a recombinant LRR-1/ELC-1/ELB-1 trimeric complex or with a similar complex containing a truncated LRR-1 protein lacking the substrate binding interface. As shown in Figure 5B, endogenous HTP-3, but not MUS-101, was specifically retained on the trimeric complex containing full length LRR-1, but not on the complex containing the truncated form of LRR-1. Taken together, these results indicate that LRR-1 physically interacts with HTP-3 and regulates its levels in the mitotic zone of the germline, suggesting that HTP-3 is likely a direct target of the CRL2^LRR-1^ E3-ligase.

#### CRL2^LRR-1^ acts through HTP-3 to control HIM-3 loading on chromosomes

Our observations indicate that CRL2^LRR-1^ and the proteasome regulate HTP-3 stability in the mitotic zone of the germline, possibly to prevent entry into meiosis. To further investigate this possibility, we asked whether HTP-3 accumulation upon inactivation of *cul-2*, *lrr-1* or the proteasome could force ectopic proliferative cells to enter meiosis.

GLP-1 promotes the proliferative fate, and the meiotic promoting factors GLD-1, NOS-3, GLD-2 and GLD-3 act downstream of Notch/GLP-1, in two parallel pathways (GLD-1/NOS-3 and GLD-2/GLD-3), to promote entry into meiosis. Simultaneous loss of both pathways causes a defect in meiotic entry that leads to germline overproliferation and prevention of gamete production [44]–[46]. Therefore, we asked whether loss of CUL-2 would cause ectopic proliferative germ cells to enter into meiosis in *gld-3 nos-3* double mutants. As a control for this experiment, we depleted CYE-1 (Cyclin E in *C. elegans*) because it has been shown recently that CYE-1 depletion forces ectopic proliferative germ cells to load HIM-3 and SUN-1 Ser8-Pi (P-SUN-1) and to enter meiosis [47].

**Figure 6:**
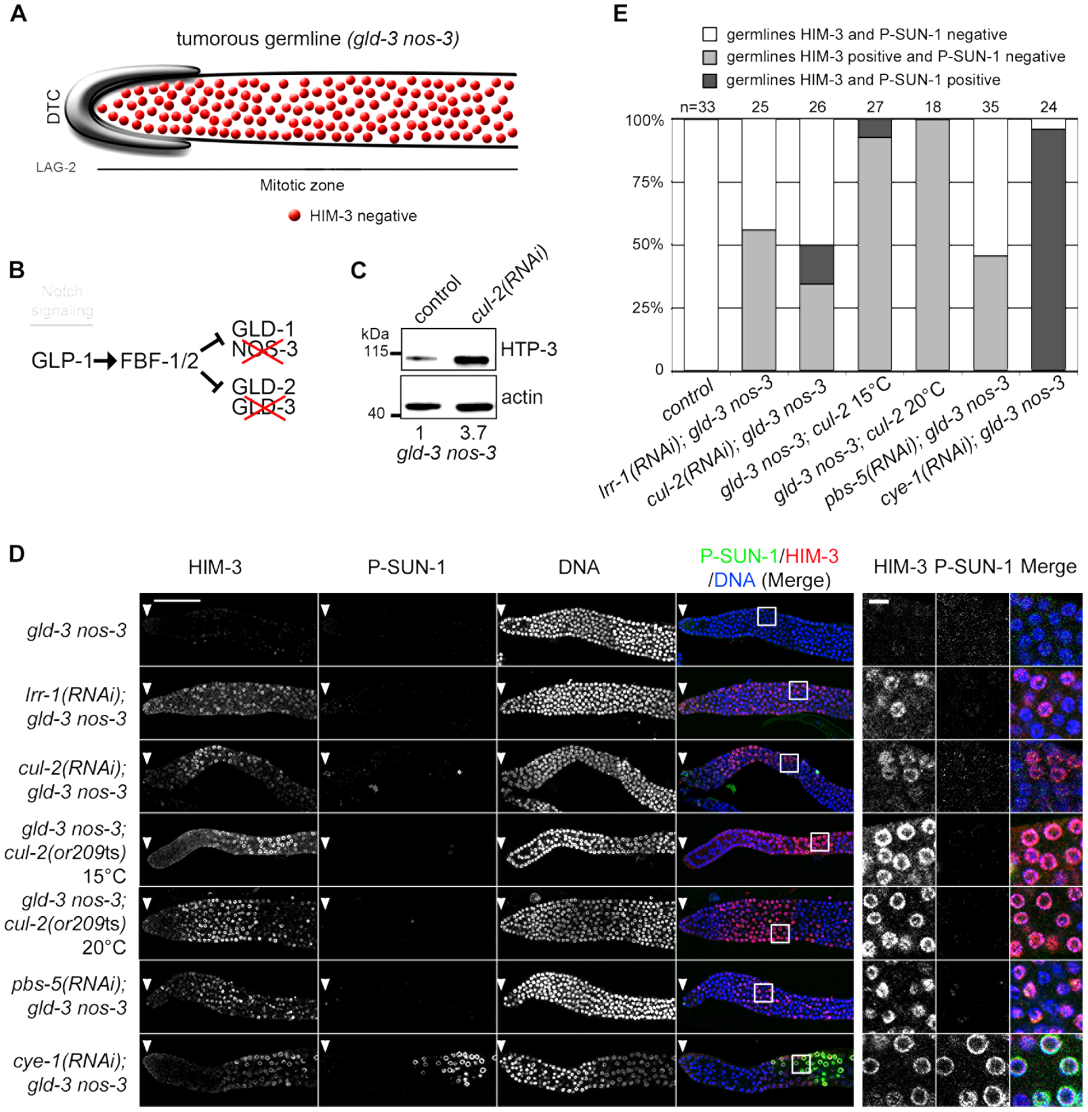
CRL2^LRR-1^ E3-Ligase controls HIM-3 loading on chromosomes. A- Schematic drawing showing that mitotic germ cells proliferate throughout the germline with no sign of meiotic entry in the *gld-3 nos-3* double mutant germlines (upper panel). B- A simplified pathway with key regulators of the mitosis/meiosis decision is shown in the lower panel. C- HTP-3 accumulates in *gld-3 nos-3* tumorous germlines upon *cul-2(RNAi)*. Total *gld-3 nos-3* worm extracts from *control* or *cul-2(RNAi)* were separated by SDS-PAGE and immunoblotted with HTP-3 (upper panel) and actin (lower panel) antibodies (loading control). The value at the bottom is the ratio between HTP-3 and actin signal intensities. The wild-type value was arbitrary defined as 1. D- Representative images of dissected gonads, of the indicated genotypes stained with HIM-3 (red) and SUN-1 Ser8-Pi (P-SUN-1, green) antibodies and with DAPI (blue). RNAi treatments were performed from the L1 (control, *cul-2*, *lrr-1*, *cye-1*) or L3 (*pbs-5*) stage at 20°C. Germlines in each panel were treated identically and fluorescent images taken at the same settings. Scale bars: 50 µM. The boxed regions, encompassing representative nuclei, are shown at higher magnification on the right panels. Scale bar: 5 µm. E- Graph showing the percentage of tumorous *gld-3 nos-3* germlines HIM-3 and SUN-1 Ser8-Pi negative (white bars), HIM-3 positive and P-SUN-1 negative (light grey), and both, HIM-3 and P-SUN-1 positive (dark grey), as scored by counting the number of nuclei rows that contain at least 5 gcd with cells expressing HIM-3 and P-SUN-1 positive.

As shown in the Figure 6C, HTP-3 accumulates in tumorous *gld-3 nos-3* germlines upon inactivation of *cul-2*, as revealed by western blot analysis. We then used immunofluorescence to test whether this accumulation is accompanied by a change in germ cell fate by monitoring the accumulation of the HIM-3 and P-SUN-1 meiotic markers. *gld-3 nos-3* germlines inactivated with control dsRNA displayed ectopic proliferating germ cells with no sign of meiotic entry as revealed by the absence of HIM-3 and P-SUN-1 meiotic markers (Figure 6B and 6C). By contrast, RNAi-mediated depletion of *cul-2*, *lrr-1* or *pbs-5* forced many germ cells to express HIM-3 to high levels. However, these germ cells remain mitotic because they do not express the other meiotic marker P-SUN-1 to significant levels. Similar results were obtained using the *cul-2*; *gld-3 nos-3* triple mutant (Figure 6B and 6C). As reported previously, CYE-1 depletion causes *gld-3 nos-3* germ cells to enter into meiosis as revealed by the accumulation of HIM-3 and P-SUN-1 meiotic markers.

Collectively, these results indicate that CUL-2 and LRR-1 inhibit only the first step of meiotic prophase by controlling HTP-3 stability and HIM-3 recruitment, whereas the CYE-1/CDK-2 kinase has a broader function in preventing meiotic entry.

## Discussion

Through the isolation and characterization of a unique temperature-sensitive mutant allele of the *cul-2* gene, we have shown that CUL-2 plays a critical role in germline development in *C. elegans*. In particular, CUL-2, in combination with LRR-1, i) promotes germ cell proliferation, ii) participates in the proliferation versus meiotic entry decision and iii) inhibits progression through the first step of meiotic prophase by regulating the stability of the axial element HTP-3.

### *cul-2*(*or209*ts) mutant: A sensitized genetic background to analyze CRL2 functions during *C. elegans* development

Temperature-sensitive (ts) alleles have been instrumental for discovering the function of essential genes in *C. elegans*, in particular for genes like CUL-2 with roles in multiple processes. Indeed, ts alleles present numerous advantages: they often only partially reduce gene function even at the fully restrictive temperature, and they allow for modulation of activity through analysis at multiple temperatures.

CUL-2 is an essential gene in *C. elegans* that is highly expressed in the germline and in the early embryo where it has been implicated in numerous processes. Our phenotypic analysis revealed that the *cul-2*(*or209*ts) mutant recapitulates all the *cul-2* loss of function phenotypes including feminization of the germline (Figure 1F), defects in cell fate determination with PIE-1 accumulation in somatic blastomeres (Figure S2), and defects in cell cycle progression in the early embryo [24] (data not shown). The penetrance of each phenotype varies with the duration of the shift at restrictive temperature, and thus this *cul-2*(*or209*ts) mutation provides a useful sensitized genetic background for identifying new *in vivo* functions of CRL2 complexes and, most importantly, for identifying its targets. Finally, other temperature-sensitive mutants affecting the ubiquitin-proteolytic system have been identified in *C. elegans* [48]–[50], but *cul-2*(*or209*ts) is to our knowledge the first conditional mutation reported for a metazoan cullin.

### CRL2^LRR-1^ regulates germ cell proliferation by counteracting the DNA replication checkpoint

Using the *cul-2*(*or209*ts) allele, we confirmed our previous observations indicating that loss of the CRL2^LRR-1^ E3-ligase causes hyperactivation of the ATL-1/DNA replication checkpoint in germ cells, resulting in a cell cycle arrest and adult sterility [19]. Germ cell nuclei were enlarged and reduced in number in the *cul-2*(*or209*ts) mutant at 25°C, a phenotype that was partially suppressed by RNAi-mediated inactivation of *atl-1* (Figure 2). Furthermore, reducing *mus-101* and *atl-1* function by RNAi increased the hatching rate of the *cul-2*(*or209*ts) mutant (Figure 5), further demonstrating that loss of CRL2^LRR-1^ function triggers activation of the ATL-1/DNA replication checkpoint pathway.

Why is the ATL-1 checkpoint pathway hyperactivated in *cul-2*(*or209*ts) and *lrr-1(0)* mutants? We believe that one function of the CRL2^LRR-1^ complex is to regulate DNA replication integrity, both in germ cells and in early embryos [19]. The ATL-1 pathway is thus activated primarily in response to DNA replication defects in the *lrr-1(0)* [19] and *cul-2*(*or209*ts) mutants (this study). In addition, our observations suggest that the inappropriate accumulation of structural components of meiotic chromosomes in *lrr-1(0)* mutants may also contribute to the activation of the ATL-1 checkpoint pathway (see below).

### CUL-2 participates in the proliferation versus meiotic entry decision

Besides promoting germ cell proliferation, our results suggest that CUL-2 influences the balance between stem cell self-renewal and meiotic differentiation possibly by regulating the stability of meiotic promoting factors. In the *C. elegans* germline, GLP-1/Notch signaling controls the decision between self-renewal and entry into the meiotic cell cycle [28]. Downstream of Notch, the RNA-binding proteins FBF-1/2 prevent meiotic entry by repressing the translation of meiotic promoting factors, and, of structural components of meiotic chromosomes [32],[33],[51]. In addition to this regulatory network, which is largely translational in nature, there is increasing evidence that post-translational regulations play also a critical role in regulating the proliferation versus meiotic entry decision. For instance, the Cyclin E/Cdk2 kinase acts with the Notch pathway to promote the proliferative fate and to prevent meiotic entry [47], [52], and our results indicate that CUL-2 plays also a role in this process. Indeed, the *cul-2ts* mutant enhanced the premature meiotic entry phenotype of the *glp-1(bn18)* mutant and when combined with the *cul-2ts* mutant, both *fbf-1* and *fbf-2* mutants showed a smaller mitotic zone than single mutants (Figure 3). This function of CUL-2 in influencing the balance between germ cell self-renewal and meiotic entry is likely independent of the DNA replication checkpoint pathway given that *atl-1* depletion does not affect the size of the mitotic zone (Figure 2).

These observations suggest the existence of a complex network of post-transcriptional and post-translational regulatory mechanisms to regulate the balance between germ cell self-renewal and meiotic entry.

What is the role of CUL-2 in this network? CUL-2 might act independently at multiple levels, for instance by regulating the activity of the CYE-1/CDK-2 kinase, and by regulating the stability both of meiotic promoting factors and of structural components of meiotic chromosomes in the mitotic zone of the germline. A gradient of high/low CYE-1/CDK-2 kinase is established along the distal to proximal end of the germline, and this gradient appears important for the self-renewal versus meiotic entry decision [47], [52]. Three complementary mechanisms establish this gradient: in the meiotic zone i) GLD-1 inhibits CYE-1 translation [53], ii) CYE-1 subunit is targeted for degradation by an E3-ligase nucleated around CUL-1 [47] and iii) the cyclin-dependent kinase inhibitor CKI-2 accumulates and inhibits CYE-1/CDK-2 activity [53]. In the mitotic zone, CKI-2 translation is inhibited by Notch and FBF-1/2. CKI-2 is not essential for germ cell proliferation but plays a role in the maintenance of the germline by influencing the proliferation versus meiotic entry decision [54]. CUL-2 has been implicated in the regulation of CKIs stability [18] and thus CUL-2 might target CKI-2 for degradation in the mitotic zone of the germline to maintain high CYE-1/CDK-2 activity in this region.

Alternatively, CUL-2 might act with CYE-1/CDK-2 to control the stability of meiotic promoting factors in the mitotic zone of the germline. Consistent with this hypothesis, substrate phosphorylation often is a pre-requisite for recognition by an E3 ligase [15], and it has been shown recently that CYE-1/CDK-2 phosphorylates GLD-1 and thereby regulates its stability in the mitotic zone of the germline [52], suggesting that CUL-2 might regulate GLD-1 degradation in germline stem cells. However, we failed to detect significant GLD-1 accumulation in germ cells located in the most distal part of the germline upon inactivation of *cul-2* (data not shown), suggesting that CUL-2 might not be involved in GLD-1 degradation. Nevertheless, we cannot exclude the possibility that a small fraction of GLD-1 accumulates upon inactivation of *cul-2* and thereby contributes to the *cul-2* phenotype.

### CRL2^LRR-1^ acts through HTP-3 to inhibit assembly of the synaptonemal complex

**Figure 7:**
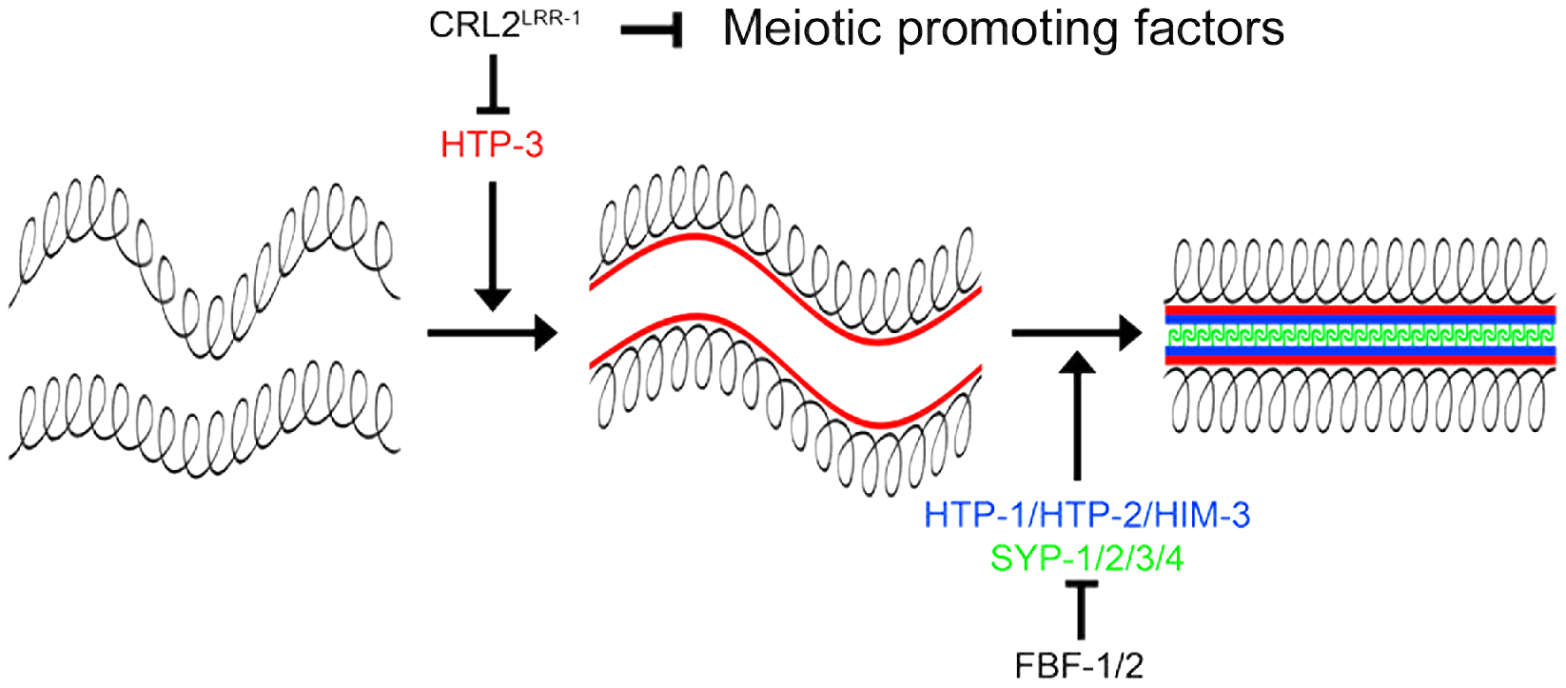
CRL2^LRR-1^ regulates entry into meiosis. The axial element HTP-3 (red) promotes the assembly of the synaptonemal complex by triggering the recruitment of the other axial element components HIM-3, HTP-1, HTP-2 (bleu) and the transverse filaments (SYP-1/2/3/4) (green). CRL2^LRR-1^ inhibits the accumulation of HTP-3 whereas FBF-1/2 prevent the expression of structural components of meiotic chromosomes in the mitotic region of the germline. CRL2^LRR-1^ also acts independently of HTP-3 to prevent meiotic entry presumably by controlling the stability of unknown meiotic promoting factor(s).

Although the mechanisms by which CUL-2 influences the proliferation versus meiotic entry decision remain to be identified, our results indicate that CRL2^LRR-1^ negatively regulates progression through meiotic prophase by controlling the stability of HTP-3 (Figure 7). HTP-3 accumulates in germ cells upon inactivation of *cul-2*, *lrr-1* and the proteasome, and HTP-3 physically interacts with LRR-1 *in vitro*. Furthermore, reducing *htp-3* function, using the *htp-3(vc75)* allele or with RNAi, suppressed *lrr-1(tm3543)* sterility and increased the hatching rate of the *cul-2*(*or209*ts) mutant, respectively. In addition, reducing *syp-1* function also increased the hatching rate of the *cul-2*(*or209*ts) mutant. These results suggest that premature accumulation of HTP-3 on chromosomes allows for inappropriate HIM-3 and subsequently SYP-1 binding. This inappropriate assembly of SC components on chromosomes likely contributes to DNA replication checkpoint activation. However, although SC components appear to be expressed and loaded inappropriately on chromosomes upon inactivation of the CRL2^LRR-1^ E3-Ligase, germ cells do not appear to enter into meiosis because we failed to detect SUN-1 Ser8-Pi on chromosomes of ectopic proliferating germ cells upon inactivation of *cul-2* or *lrr-1*. This observation indicates that HTP-3 accumulation is not sufficient to drive progression through meiotic prophase and that CUL-2 acts independently of HTP-3 to influence to proliferation versus meiotic entry decision, presumably by controlling the stability of meiotic regulator(s) in the mitotic zone of the germline (Figure 7).

In conclusion, our results indicate that CUL-2 acts at multiple levels in the germline to coordinate germ cell proliferation and meiotic entry and thus emerged as a critical regulator of germline development in *C. elegans*.

## Materials and Methods

### Nematode strains, strain construction, and culture conditions

*C. elegans* strains were cultured and maintained using standard procedures [55]. Strains of the following genotypes were used: N2 Bristol (wild type); *lrr-1(tm3543)II/mIn1[mIs14 dpy-10(e128)]II* [19]; *cul-2*(*or209*ts)*III* [24]; *htp-3(vc75)I* [42]; *htp-3(vc75)I*; *lrr-1(tm3543)II/mIn1[mIs14 dpy-10(e128)]II* (this study); *glp-1(bn18)* [34]; *atl-1(tm853)IV* [56]; *gld-3(q730) nos-3(q650)II/mIn1[mIs14 dpy-10(e128)]II* [57]; *gld-3(q730) nos-3(q650)II/mIn1[mIs14 dpy-10(e128)]II; cul-2*(*or209*ts)*III* (this study), *fbf-1(ok91)II* [32], *glp-1(bn18) cul-2*(*or209*ts)*III* (this study); *fbf-1(ok91)II*; *cul-2*(*or209*ts) *III* (this study); *fbf-2(q738)II* [58], *fbf-2(q738)II*; *cul-2*(*or209*ts)*III* (this study).

### Single nucleotide polymorphism (SNP) mapping and pyrosequencing

For this study, *or209* worms were mated to the Hawaiian CB4856 *C. elegans* wild isolate, and recombinant F2 lines homozygous for the *or209* mutation were isolated based on embryonic lethality at the restrictive temperature. Several SNPs located between −27.2 cM and 21.25 cM along the third chromosome were amplified by PCR from these recombinants, essentially as described [59], and genotyped using pyrosequencing technology. Briefly, PCR amplifications were performed from single worms using Taq DNA polymerase (New England Biolabs) and specific primers for each SNP. The purification of single-stranded PCR amplicons and the pyrosequencing reactions were subsequently performed according to manufacturer's instructions using a Pyromark Q96 ID instrument (Biotage). This analysis positioned the *or209* mutation between +18.52 cM and the right end of chromosome III. This region contains *cul-2*, and we showed that all embryos from *or209/cul-2(ek1)* trans-heterozygotes (*ek1* is a null allele of *cul-2* [18]) failed to hatch.

### RNA interference (RNAi)

*Escherichia coli* clones expressing dsRNA to deplete *C. elegans* genes were obtained from the MRC Geneservice (Cambridge, U) [60], [61]. RNAi feeding was performed as described using 2 mM IPTG (RNAi plates) [62].

### Statistical analysis

The results are presented as means ± S.E.M. In all graphs, data were compared by a Mann-Whitney test (two-tailed p) or Student test (Figure 3 and Figure 5). All calculations were performed with InStat3 software (Graphpad). * p<0.05; ** p<0.01; *** p<0.001.

### Hydroxyurea treatment

L4 animals were fed on NGM plates containing 10 mM final HU prior to analysis. Germlines were then dissected and stained with DAPI. Quantification of HU-induced cell cycle arrest was performed by counting the number of nuclei in a defined volume (20 000 µm^3^).

### Microscopy, immunochemistry, and fluorescence microscopy

Germlines were dissected in PBS followed by freeze-crack, immersion in cold MeOH (−20°C) for 1 min, and fixation in 1× PBS, 0.08 M HEPES (pH 6.9), 1.6 mM MgSO_4_, 0.8 mM EGTA and 3.7% paraformaldehyde for 30 min in a humidity chamber at room temperature. Slides were washed 3×5 min, blocked for 1 h in PBT (1× PBS, 0.1% Triton X-100, and 5% BSA), and incubated overnight at 4°C with primary antibodies diluted in PBT. Working dilutions for the primary antibodies were 1∶1000 for rabbit anti-HIM-3 (M. Zetka), 1∶100 for rabbit anti-HTP-3 (M. Zetka) and 1∶1000 anti-SUN-1 Ser8-Pi (V. Jantsch). Slides were later incubated for 30 min at room temperature with secondary antibodies coupled to the Alexa 488 and 568 fluorophore (1∶600, Molecular Probes). Next, germlines were mounted in Vectashield Mounting Medium with DAPI (Vector). Fixed germlines were imaged using either a TCS SP5 confocal microscope (Leica) or a LSM 710 confocal microscope (Zeiss) with 40× objectives. Confocal images correspond to the projection of confocal Z-stacks spanning maximum 5 µm. Captured images were processed using ImageJ and Adobe Photoshop.

For whole-worm images, worms were immobilised with 20 mM levamisole and mounted on 2% agarose pads. Images were then acquired using an Axiovert 200 inverted microscope equipped with DIC optics.

### Protein extracts and antibodies

Standard procedures were used for SDS–PAGE and western blotting. The following antibodies were used in this study: primary antibodies were directed against CUL-2 [19], ELC-1 [19], HTP-3 (M. Zetka), HIM-3 (M. Zetka), SUN-1 Ser8-Pi [35], Tubulin (Sigma), Actin (Sigma), TRA-1 [63] and MUS-101 [36]; secondary antibodies conjugated to peroxidase against rabbit or mouse were purchased from Sigma.

LRR-1/ELC-1/ELB-1 trimeric complexes were expressed in *E. coli* and purified as described [19].

Total worm extracts were prepared by cryolysis, as previously described [64], and loaded onto trimeric complexes immobilised on T7-agarose beads (Novagen) for 2 hours at 4°C. After five washes, proteins were eluted with sample buffer and separated by SDS-PAGE.

## Supporting Information

Figure S1 Identification of a temperature-sensitive allele in the *cul-2* gene. A- Flow chart of the approach used to screen temperature-sensitive mutants affecting the function of the CRL2^LRR-1^ complex. The basic idea is to screen for mutants that behave like *lrr-1(0)* mutant animals. *lrr-1(0)* animals are sterile with a small germline but this phenotype is fully suppressed by inactivation of the ATL-1/DNA replication checkpoint pathway. Furthermore, in the early embryo, depletion of *lrr-1* by RNAi causes a specific delay in the division of the P1 germline blastomere (*Div* phenotype for cell division defective). Therefore, we screened a collection of temperature-sensitive *div* mutants that are sterile at 25°C but recover fertility upon inactivation of the ATL-1 checkpoint pathway. The *div* mutants were isolated essentially as described. *div(or)* mutant L1 larvae were fed with control or *atl-1* dsRNA at restrictive temperature (25°C) until adulthood and the percentage of fertile animals was determined by DIC microscopy. B- Graph showing the percentage of fertile *div(or)* mutants after control (dark bars) or *atl-1*(RNAi) (grey bars). C-Mapping of *div(or209ts)* mutation to the end of chromosome III is shown. The ratio of *or209* N2 DNA *(blue bars)* to “Hawaiian” DNA (orange bars) measured at various single nucleotide polymorphisms (SNPs; −27.2, −9, −3.9, 0.6, 4, 8.15, 13.29, 15.87, 18.52, 20.6, 21.25 centimorgan (cM)) are presented on the graph; *cul-2* localised at 21.36 cM. D- Embryonic lethality and brood size of *cul-2*(*or209*ts) mutant animals. Graphs show the embryonic viability (left panel) and brood size (right panel) of WT (dark bars) and *cul-2*(*or209*ts) (grey bars) animals at permissive temperature (15°C).
(TIF)

Figure S2 Mislocalisation of PIE-1 in *cul-2*(*or209*ts) embryos. Fluorescent micrographs of *cul-2*(*or209*ts) embryos expressing GFP::PIE-1 are shown. Note the mislocalisation of GFP::PIE-1 to somatic blastomeres in *cul-2*(*or209*ts) embryos produced at 25°C (right panels).
(TIF)

Figure S3 The ATL-1/DNA replication checkpoint pathway is hyperactivated in *lrr-1(tm3543)*; *htp-3(vc75)* double mutants. A- Lineage diagram on the right, vertical bars indicate time, horizontal bars indicate cell division, anterior daughters are positioned to the left, and posterior daughters to the right. B- The elapsed time, in seconds (s), between AB and P1 cytokinesis was determined and plotted. Note that the ATL-1 checkpoint pathway is hyperactivated in *lrr-1(tm3543)*; *htp-3(vc75)* double mutant embryos as revealed by a severe delay in the division of the posterior P1 blastomere in two-cell stage embryos.
(TIF)

Table S1 List of *div* alleles.
(TIF)

## References

[1] Hershko A, Ciechanover A (1998) The ubiquitin system. Annu Rev Biochem 67: 425–479. 9759494 10.1146/annurev.biochem.67.1.425

[2] Shabek N, Ciechanover A (2010) Degradation of ubiquitin: the fate of the cellular reaper. Cell Cycle 9: 523–530. 20107325 10.4161/cc.9.3.11152

[3] Hershko A, Heller H, Elias S, Ciechanover A (1983) Components of ubiquitin-protein ligase system. Resolution, affinity purification, and role in protein breakdown. J Biol Chem 258: 8206–8214. 6305978

[4] Welcker M, Clurman BE (2008) FBW7 ubiquitin ligase: a tumour suppressor at the crossroads of cell division, growth and differentiation. Nat Rev Cancer 8: 83–93. 18094723 10.1038/nrc2290

[5] Frescas D, Pagano M (2008) Deregulated proteolysis by the F-box proteins SKP2 and beta-TrCP: tipping the scales of cancer. Nat Rev Cancer 8: 438–449. 18500245 10.1038/nrc2396 PMC2711846

[6] Lee J, Zhou P (2012) Pathogenic Role of the CRL4 Ubiquitin Ligase in Human Disease. Front Oncol 2: 21. 22649780 10.3389/fonc.2012.00021 PMC3355902

[7] Ye Y, Rape M (2009) Building ubiquitin chains: E2 enzymes at work. Nat Rev Mol Cell Biol 10: 755–764. 19851334 10.1038/nrm2780 PMC3107738

[8] Chau V, Tobias JW, Bachmair A, Marriott D, Ecker DJ, et al (1989) A multiubiquitin chain is confined to specific lysine in a targeted short-lived protein. Science 243: 1576–1583. 2538923 10.1126/science.2538923

[9] Thrower JS, Hoffman L, Rechsteiner M, Pickart CM (2000) Recognition of the polyubiquitin proteolytic signal. EMBO J 19: 94–102. 10619848 10.1093/emboj/19.1.94 PMC1171781

[10] Glotzer M, Murray AW, Kirschner MW (1991) Cyclin is degraded by the ubiquitin pathway. Nature 349: 132–138. 1846030 10.1038/349132a0

[11] Jin L, Williamson A, Banerjee S, Philipp I, Rape M (2008) Mechanism of ubiquitin-chain formation by the human anaphase-promoting complex. Cell 133: 653–665. 18485873 10.1016/j.cell.2008.04.012 PMC2696189

[12] Mocciaro A, Rape M (2012) Emerging regulatory mechanisms in ubiquitin-dependent cell cycle control. J Cell Sci 125: 255–263. 22357967 10.1242/jcs.091199 PMC3283867

[13] Deshaies RJ, Joazeiro CA (2009) RING domain E3 ubiquitin ligases. Annu Rev Biochem 78: 399–434. 19489725 10.1146/annurev.biochem.78.101807.093809

[14] Sarikas A, Hartmann T, Pan ZQ (2011) The cullin protein family. Genome Biol 12: 220. 21554755 10.1186/gb-2011-12-4-220 PMC3218854

[15] Petroski MD, Deshaies RJ (2005) Function and regulation of cullin-RING ubiquitin ligases. Nat Rev Mol Cell Biol 6: 9–20. 15688063 10.1038/nrm1547

[16] Merlet J, Burger J, Gomes JE, Pintard L (2009) Regulation of cullin-RING E3 ubiquitin-ligases by neddylation and dimerization. Cell Mol Life Sci 10.1007/s00018-009-8712-7 PMC11115538 19194658

[17] Okumura F, Matsuzaki M, Nakatsukasa K, Kamura T (2012) The Role of Elongin BC-Containing Ubiquitin Ligases. Front Oncol 2: 10. 22649776 10.3389/fonc.2012.00010 PMC3355856

[18] Feng H, Zhong W, Punkosdy G, Gu S, Zhou L, et al (1999) CUL-2 is required for the G1-to-S-phase transition and mitotic chromosome condensation in *Caenorhabditis elegans* . Nat Cell Biol 1: 486–492. 10587644 10.1038/70272

[19] Merlet J, Burger J, Tavernier N, Richaudeau B, Gomes JE, et al (2010) The CRL2LRR-1 ubiquitin ligase regulates cell cycle progression during C. elegans development. Development 137: 3857–3866. 20978077 10.1242/dev.054866 PMC3188596

[20] Starostina NG, Simpliciano JM, McGuirk MA, Kipreos ET (2010) CRL2(LRR-1) targets a CDK inhibitor for cell cycle control in C. elegans and actin-based motility regulation in human cells. Dev Cell 19: 753–764. 21074724 10.1016/j.devcel.2010.10.013 PMC3053091

[21] Sonneville R, Gonczy P (2004) Zyg-11 and cul-2 regulate progression through meiosis II and polarity establishment in C. elegans. Development 131: 3527–3543. 15215208 10.1242/dev.01244

[22] Liu J, Vasudevan S, Kipreos ET (2004) CUL-2 and ZYG-11 promote meiotic anaphase II and the proper placement of the anterior-posterior axis in *C. elegans* . Development 131: 3513–3525. 15215209 10.1242/dev.01245

[23] DeRenzo C, Reese KJ, Seydoux G (2003) Exclusion of germ plasm proteins from somatic lineages by cullin-dependent degradation. Nature 424: 685–689. 12894212 10.1038/nature01887. PMC1892537

[24] Encalada SE, Martin PR, Phillips JB, Lyczak R, Hamill DR, et al (2000) DNA replication defects delay cell division and disrupt cell polarity in early Caenorhabditis elegans embryos. Dev Biol 228: 225–238. 11112326 10.1006/dbio.2000.9965

[25] Starostina NG, Lim JM, Schvarzstein M, Wells L, Spence AM, et al (2007) A CUL-2 ubiquitin ligase containing three FEM proteins degrades TRA-1 to regulate C. elegans sex determination. Dev Cell 13: 127–139. 17609115 10.1016/j.devcel.2007.05.008 PMC2064902

[26] Byrd DT, Kimble J (2009) Scratching the niche that controls Caenorhabditis elegans germline stem cells. Semin Cell Dev Biol 20: 1107–1113. 19765664 10.1016/j.semcdb.2009.09.005 PMC2820558

[27] Hansen D, Schedl T (2006) The regulatory network controlling the proliferation-meiotic entry decision in the Caenorhabditis elegans germ line. Curr Top Dev Biol 76: 185–215. 17118267 10.1016/S0070-2153(06)76006-9

[28] Kimble J, Crittenden SL (2007) Controls of germline stem cells, entry into meiosis, and the sperm/oocyte decision in Caenorhabditis elegans. Annu Rev Cell Dev Biol 23: 405–433. 17506698 10.1146/annurev.cellbio.23.090506.123326

[29] Zetka MC, Kawasaki I, Strome S, Muller F (1999) Synapsis and chiasma formation in Caenorhabditis elegans require HIM-3, a meiotic chromosome core component that functions in chromosome segregation. Genes Dev 13: 2258–2270. 10485848 10.1101/gad.13.17.2258 PMC317003

[30] MacQueen AJ, Villeneuve AM (2001) Nuclear reorganization and homologous chromosome pairing during meiotic prophase require C. elegans chk-2. Genes Dev 15: 1674–1687. 11445542 10.1101/gad.902601 PMC312723

[31] Vasudevan S, Starostina NG, Kipreos ET (2007) The Caenorhabditis elegans cell-cycle regulator ZYG-11 defines a conserved family of CUL-2 complex components. EMBO Rep 8: 279–286. 17304241 10.1038/sj.embor.7400895 PMC1808032

[32] Crittenden SL, Bernstein DS, Bachorik JL, Thompson BE, Gallegos M, et al (2002) A conserved RNA-binding protein controls germline stem cells in Caenorhabditis elegans. Nature 417: 660–663. 12050669 10.1038/nature754

[33] Kershner AM, Kimble J (2010) Genome-wide analysis of mRNA targets for Caenorhabditis elegans FBF, a conserved stem cell regulator. Proc Natl Acad Sci U S A 107: 3936–3941. 20142496 10.1073/pnas.1000495107 PMC2840422

[34] Qiao L, Lissemore JL, Shu P, Smardon A, Gelber MB, et al (1995) Enhancers of glp-1, a gene required for cell-signaling in Caenorhabditis elegans, define a set of genes required for germline development. Genetics 141: 551–569. 8647392 10.1093/genetics/141.2.551 PMC1206755

[35] Penkner AM, Fridkin A, Gloggnitzer J, Baudrimont A, Machacek T, et al (2009) Meiotic chromosome homology search involves modifications of the nuclear envelope protein Matefin/SUN-1. Cell 139: 920–933. 19913286 10.1016/j.cell.2009.10.045

[36] Holway AH, Hung C, Michael WM (2005) Systematic, RNA-interference-mediated identification of mus-101 modifier genes in Caenorhabditis elegans. Genetics 169: 1451–1460. 15654100 10.1534/genetics.104.036137 PMC1449550

[37] MacQueen AJ, Phillips CM, Bhalla N, Weiser P, Villeneuve AM, et al (2005) Chromosome sites play dual roles to establish homologous synapsis during meiosis in C. elegans. Cell 123: 1037–1050. 16360034 10.1016/j.cell.2005.09.034 PMC4435800

[38] Goodyer W, Kaitna S, Couteau F, Ward JD, Boulton SJ, et al (2008) HTP-3 links DSB formation with homolog pairing and crossing over during C. elegans meiosis. Dev Cell 14: 263–274. 18267094 10.1016/j.devcel.2007.11.016

[39] MacQueen AJ, Colaiacovo MP, McDonald K, Villeneuve AM (2002) Synapsis-dependent and -independent mechanisms stabilize homolog pairing during meiotic prophase in C. elegans. Genes Dev 16: 2428–2442. 12231631 10.1101/gad.1011602 PMC187442

[40] Aravind L, Koonin EV (1998) The HORMA domain: a common structural denominator in mitotic checkpoints, chromosome synapsis and DNA repair. Trends Biochem Sci 23: 284–286. 9757827 10.1016/s0968-0004(98)01257-2

[41] Severson AF, Ling L, van Zuylen V, Meyer BJ (2009) The axial element protein HTP-3 promotes cohesin loading and meiotic axis assembly in C. elegans to implement the meiotic program of chromosome segregation. Genes Dev 23: 1763–1778. 19574299 10.1101/gad.1808809 PMC2720254

[42] Couteau F, Zetka M (2011) DNA damage during meiosis induces chromatin remodeling and synaptonemal complex disassembly. Dev Cell 20: 353–363. 21397846 10.1016/j.devcel.2011.01.015

[43] Hayashi M, Chin GM, Villeneuve AM (2007) C. elegans germ cells switch between distinct modes of double-strand break repair during meiotic prophase progression. PLoS Genet 3: e191 doi:10.1371/journal.pgen.0030191. 17983271 10.1371/journal.pgen.0030191 PMC2048528

[44] Austin J, Kimble J (1987) glp-1 is required in the germ line for regulation of the decision between mitosis and meiosis in C. elegans. Cell 51: 589–599. 3677168 10.1016/0092-8674(87)90128-0

[45] Kadyk LC, Kimble J (1998) Genetic regulation of entry into meiosis in Caenorhabditis elegans. Development 125: 1803–1813. 9550713 10.1242/dev.125.10.1803

[46] Hansen D, Hubbard EJ, Schedl T (2004) Multi-pathway control of the proliferation versus meiotic development decision in the Caenorhabditis elegans germline. Dev Biol 268: 342–357. 15063172 10.1016/j.ydbio.2003.12.023

[47] Fox PM, Vought VE, Hanazawa M, Lee MH, Maine EM, et al (2011) Cyclin E and CDK-2 regulate proliferative cell fate and cell cycle progression in the C. elegans germline. Development 138: 2223–2234. 21558371 10.1242/dev.059535 PMC3091494

[48] Kurz T, Pintard L, Willis JH, Hamill DR, Gonczy P, et al (2002) Cytoskeletal regulation by the Nedd8 ubiquitin-like protein modification pathway. Science 295: 1294–1298. 11847342 10.1126/science.1067765

[49] Kulkarni M, Smith HE (2008) E1 ubiquitin-activating enzyme UBA-1 plays multiple roles throughout C. elegans development. PLoS Genet 4: e1000131 doi:10.1371/journal.pgen.1000131. 18636104 10.1371/journal.pgen.1000131 PMC2443343

[50] Macdonald LD, Knox A, Hansen D (2008) Proteasomal regulation of the proliferation vs. meiotic entry decision in the Caenorhabditis elegans germ line. Genetics 180: 905–920. 18791239 10.1534/genetics.108.091553 PMC2567390

[51] Merritt C, Seydoux G (2010) The Puf RNA-binding proteins FBF-1 and FBF-2 inhibit the expression of synaptonemal complex proteins in germline stem cells. Development 137: 1787–1798. 20431119 10.1242/dev.050799 PMC2867315

[52] Jeong J, Verheyden JM, Kimble J (2011) Cyclin E and Cdk2 control GLD-1, the mitosis/meiosis decision, and germline stem cells in Caenorhabditis elegans. PLoS Genet 7: e1001348 doi:10.1371/journal.pgen.1001348. 21455289 10.1371/journal.pgen.1001348 PMC3063749

[53] Biedermann B, Wright J, Senften M, Kalchhauser I, Sarathy G, et al (2009) Translational repression of cyclin E prevents precocious mitosis and embryonic gene activation during C. elegans meiosis. Dev Cell 17: 355–364. 19758560 10.1016/j.devcel.2009.08.003

[54] Kalchhauser I, Farley BM, Pauli S, Ryder SP, Ciosk R (2011) FBF represses the Cip/Kip cell-cycle inhibitor CKI-2 to promote self-renewal of germline stem cells in C. elegans. EMBO J 30: 3823–3829. 21822213 10.1038/emboj.2011.263 PMC3173791

[55] Brenner S (1974) The genetics of Caenorhabditis elegans. Genetics 77: 71–94. 4366476 10.1093/genetics/77.1.71 PMC1213120

[56] Garcia-Muse T, Boulton SJ (2005) Distinct modes of ATR activation after replication stress and DNA double-strand breaks in Caenorhabditis elegans. EMBO J 24: 4345–4355. 16319925 10.1038/sj.emboj.7600896 PMC1356337

[57] Eckmann CR, Crittenden SL, Suh N, Kimble J (2004) GLD-3 and control of the mitosis/meiosis decision in the germline of Caenorhabditis elegans. Genetics 168: 147–160. 15454534 10.1534/genetics.104.029264 PMC1448115

[58] Lamont LB, Crittenden SL, Bernstein D, Wickens M, Kimble J (2004) FBF-1 and FBF-2 regulate the size of the mitotic region in the C. elegans germline. Dev Cell 7: 697–707. 15525531 10.1016/j.devcel.2004.09.013

[59] Fay D, Bender A (2008) SNPs: introduction and two-point mapping. Worm Book 1–10. 18819170 10.1895/wormbook.1.93.2 PMC10083728

[60] Fraser AG, Kamath RS, Zipperlen P, Martinez-Campos M, Sohrmann M, et al (2000) Functional genomic analysis of C. elegans chromosome I by systematic RNA interference. Nature 408: 325–330. 11099033 10.1038/35042517

[61] Kamath RS, Fraser AG, Dong Y, Poulin G, Durbin R, et al (2003) Systematic functional analysis of the Caenorhabditis elegans genome using RNAi. Nature 421: 231–237. 12529635 10.1038/nature01278

[62] Kamath RS, Martinez-Campos M, Zipperlen P, Fraser AG, Ahringer J (2001) Effectiveness of specific RNA-mediated interference through ingested double-stranded RNA in Caenorhabditis elegans. Genome Biol 2: RESEARCH0002 doi:10.1186/gb-2000-2-1-research0002. 11178279 10.1186/gb-2000-2-1-research0002 PMC17598

[63] Sasagawa Y, Otani M, Higashitani N, Higashitani A, Sato K, et al (2009) Caenorhabditis elegans p97 controls germline-specific sex determination by controlling the TRA-1 level in a CUL-2-dependent manner. J Cell Sci 122: 3663–3672. 19773360 10.1242/jcs.052415

[64] Luke-Glaser S, Roy M, Larsen B, Le Bihan T, Metalnikov P, et al (2007) CIF-1, a shared subunit of the COP9/signalosome and eukaryotic initiation factor 3 complexes, regulates MEL-26 levels in the Caenorhabditis elegans embryo. Mol Cell Biol 27: 4526–4540. 17403899 10.1128/MCB.01724-06 PMC1900047

