# Supplementary figures and images for: CRL2LRR-1 E3-Ligase Regulates Proliferation and Progression through Meiosis in the Caenorhabditis elegans Germline
Source: PLoS Genet. 2013 Mar 28;9(3):e1003375. doi: 10.1371/journal.pgen.1003375 (PMC3610609; doi:10.1371/journal.pgen.1003375)

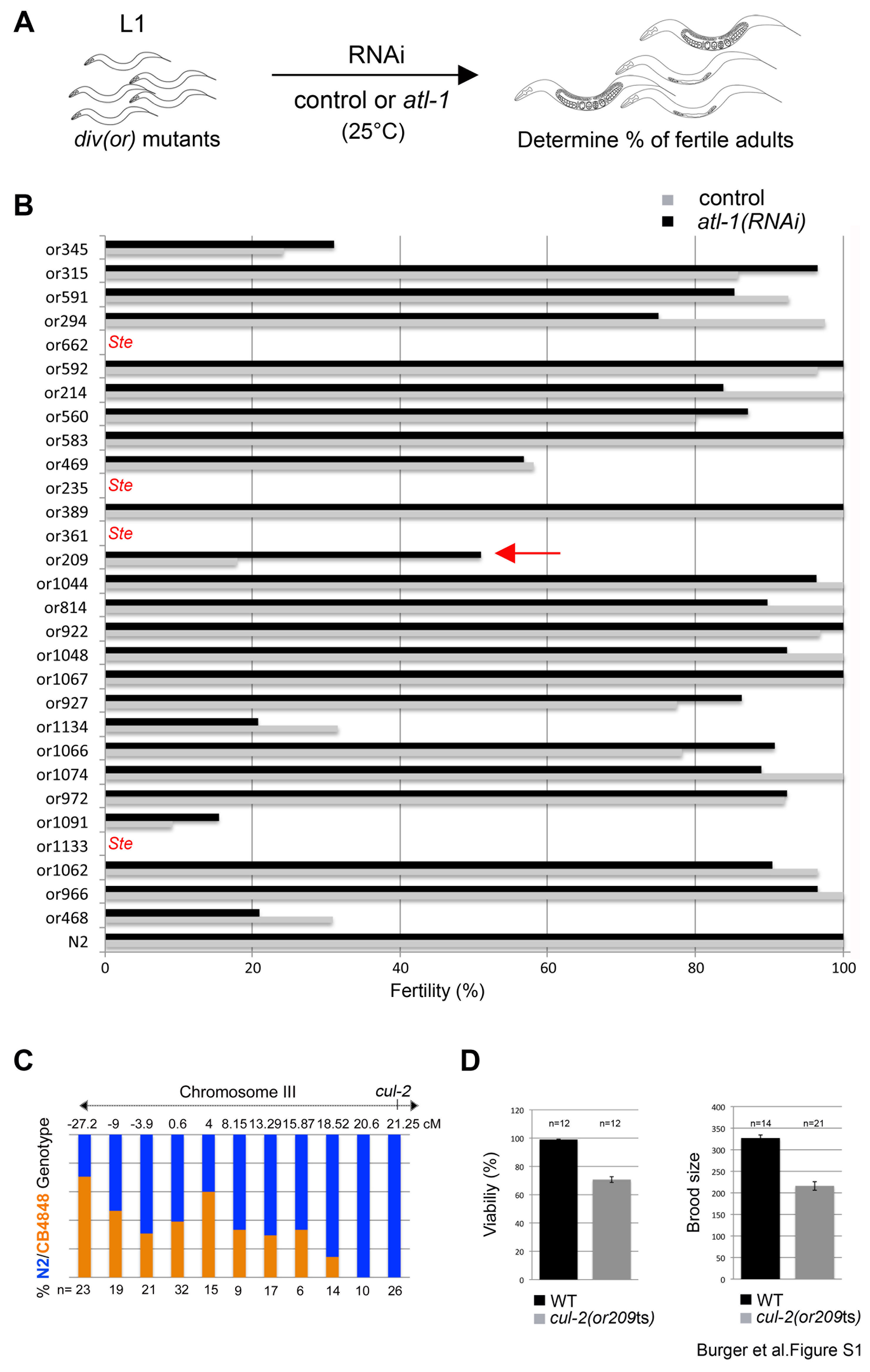

Supplement: Figure S1 — Identification of a temperature-sensitive allele in the cul-2 gene. A- Flow chart of the approach used to screen temperature-sensitive mutants affecting the function of the CRL2LRR-1 complex. The basic idea is to screen for mutants that behave like lrr-1(0) mutant animals. lrr-1(0) animals are sterile with a small germline but this phenotype is fully suppressed by inactivation of the ATL-1/DNA replication checkpoint pathway. Furthermore, in the early embryo, depletion of lrr-1 by RNAi causes a specific delay in the division of the P1 germline blastomere (Div phenotype for cell division defective). Therefore, we screened a collection of temperature-sensitive div mutants that are sterile at 25°C but recover fertility upon inactivation of the ATL-1 checkpoint pathway. The div mutants were isolated essentially as described. div(or) mutant L1 larvae were fed with control or atl-1 dsRNA at restrictive temperature (25°C) until adulthood and the percentage of fertile animals was determined by DIC microscopy. B- Graph showing the percentage of fertile div(or) mutants after control (dark bars) or atl-1(RNAi) (grey bars). C-Mapping of div(or209ts) mutation to the end of chromosome III is shown. The ratio of or209 N2 DNA (blue bars) to “Hawaiian” DNA (orange bars) measured at various single nucleotide polymorphisms (SNPs; −27.2, −9, −3.9, 0.6, 4, 8.15, 13.29, 15.87, 18.52, 20.6, 21.25 centimorgan (cM)) are presented on the graph; cul-2 localised at 21.36 cM. D- Embryonic lethality and brood size of cul-2(or209ts) mutant animals. Graphs show the embryonic viability (left panel) and brood size (right panel) of WT (dark bars) and cul-2(or209ts) (grey bars) animals at permissive temperature (15°C). (TIF) [file pgen.1003375.s001.tif]

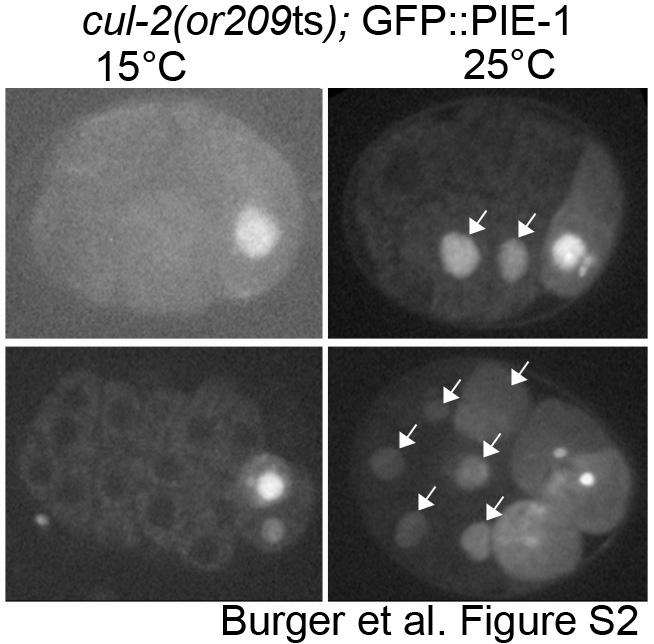

Supplement: Figure S2 — Mislocalisation of PIE-1 in cul-2(or209ts) embryos. Fluorescent micrographs of cul-2(or209ts) embryos expressing GFP::PIE-1 are shown. Note the mislocalisation of GFP::PIE-1 to somatic blastomeres in cul-2(or209ts) embryos produced at 25°C (right panels). (TIF) [file pgen.1003375.s002.tif]

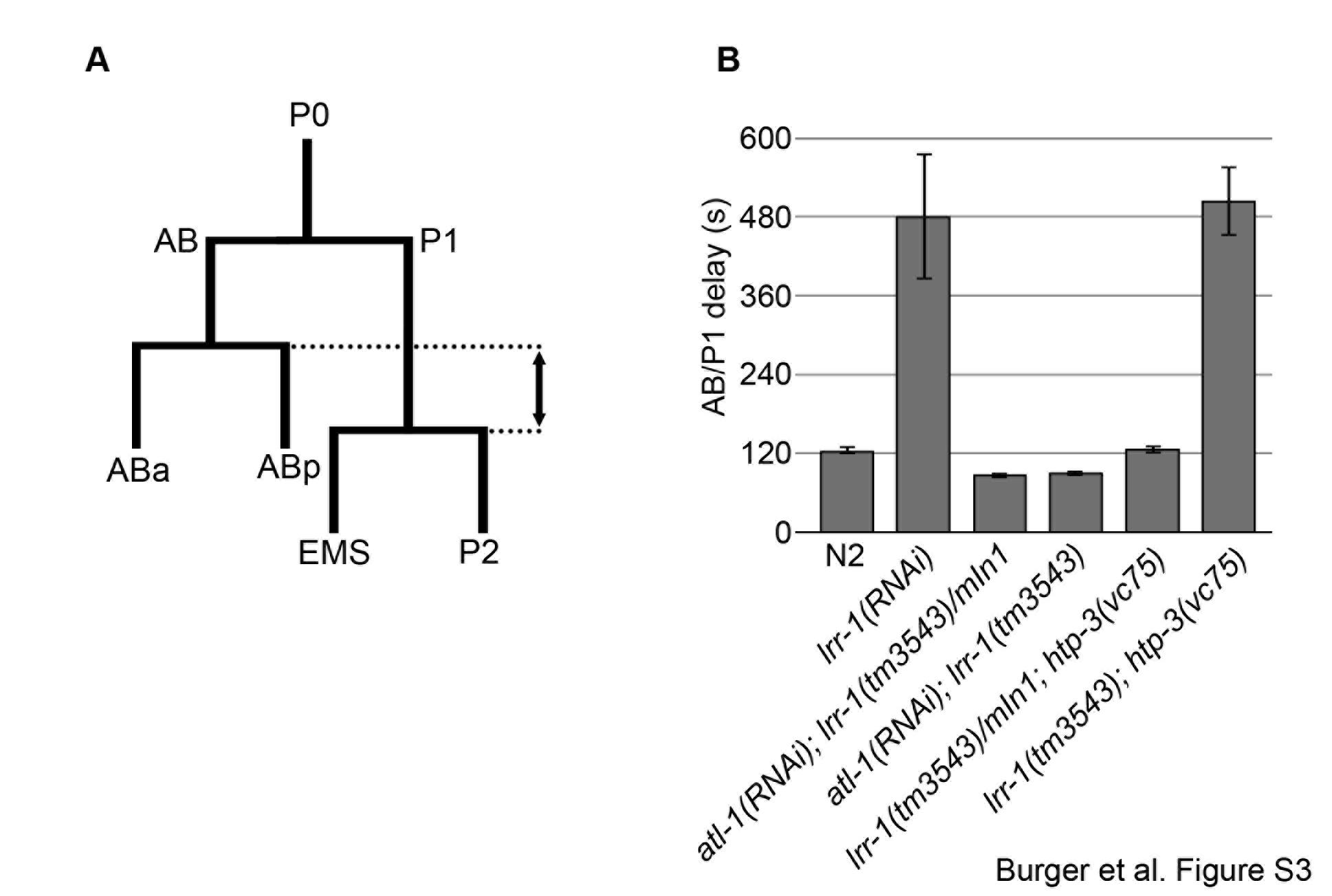

Supplement: Figure S3 — The ATL-1/DNA replication checkpoint pathway is hyperactivated in lrr-1(tm3543); htp-3(vc75) double mutants. A- Lineage diagram on the right, vertical bars indicate time, horizontal bars indicate cell division, anterior daughters are positioned to the left, and posterior daughters to the right. B- The elapsed time, in seconds (s), between AB and P1 cytokinesis was determined and plotted. Note that the ATL-1 checkpoint pathway is hyperactivated in lrr-1(tm3543); htp-3(vc75) double mutant embryos as revealed by a severe delay in the division of the posterior P1 blastomere in two-cell stage embryos. (TIF) [file pgen.1003375.s003.tif]

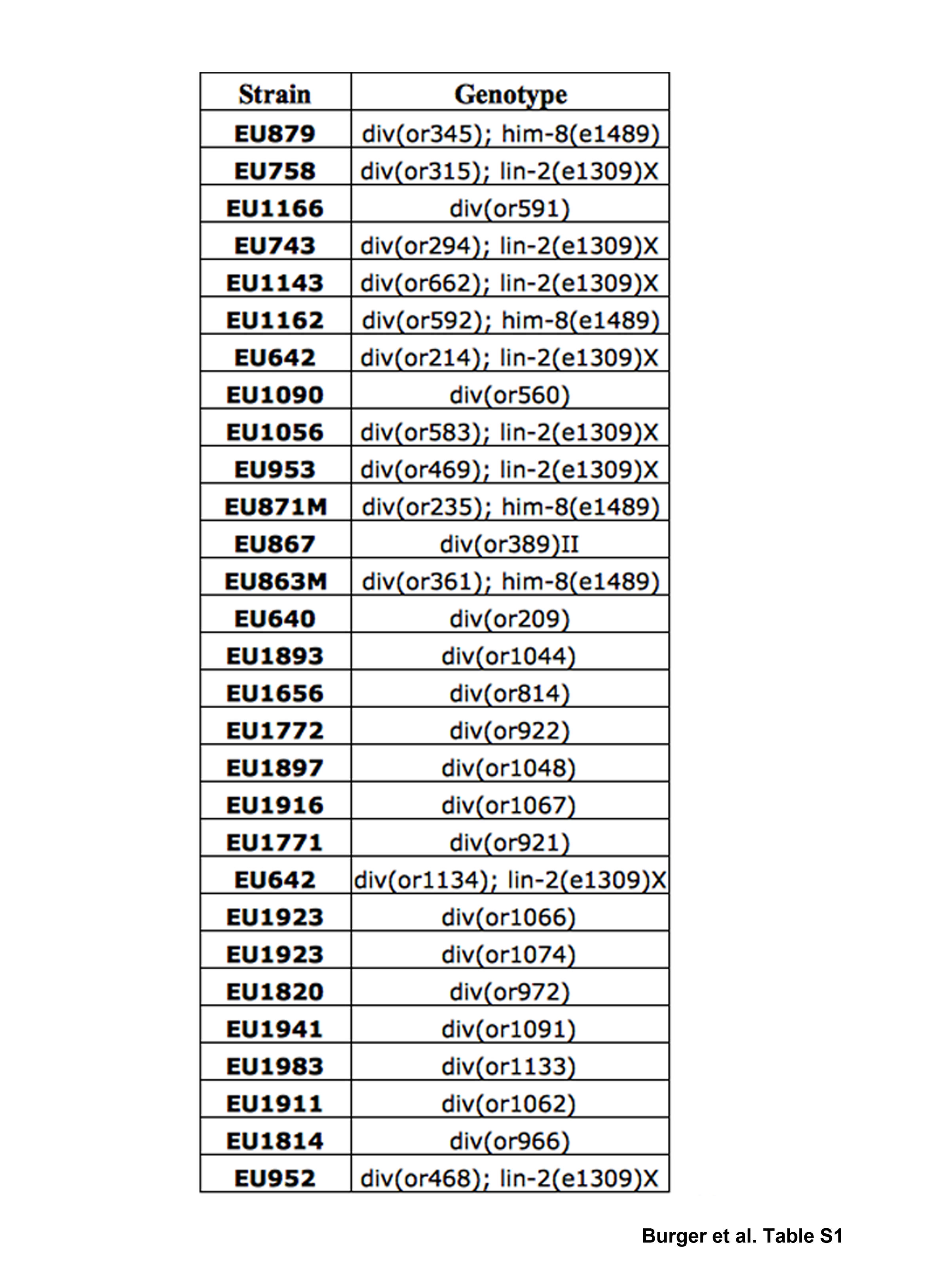

Supplement: Table S1 — List of div alleles. (TIF) [file pgen.1003375.s004.tif]
